# Field Assessment of Colorado pikeminnow Exposure to Mercury Within Its Designated Critical Habitat in Colorado, Utah, and New Mexico

**DOI:** 10.1007/s00244-018-0566-2

**Published:** 2018-09-26

**Authors:** Barbara C. Osmundson, Joel D. Lusk

**Affiliations:** 1 Colorado Ecological Services, Western Colorado Field Office, US Fish and Wildlife Service, 445 West Gunnison Ave., Suite 240, Grand Junction, CO 81501-5711 USA; 2 New Mexico Ecological Services, US Fish and Wildlife Service, 2105 Osuna Road NE, Albuquerque, NM 87113-1001 USA; 3 Present Address: 380 34 Road, Palisade, CO 81526 USA

## Abstract

Mercury contamination in freshwater fish is widespread across North America, including the western United States. Atmospheric mercury from both natural and manmade emissions deposits into watersheds and, through methylation and biomagnification, accumulates in aquatic food webs. Highest mercury concentrations are found in predatory fish. The endangered Colorado pikeminnow (*Ptychocheilus lucius*) is a long-lived, top-level piscivore endemic to the Colorado River basin. Mercury exposure to Colorado pikeminnow and another native fish species, the roundtail chub (*Gila robusta*), was assessed by analyzing muscle tissues collected using a nonlethal technique. Mercury concentrations in Colorado pikeminnow > 400-mm long, captured from critical habitat throughout the species’ present range, exceeded the tissue threshold-effect level of 0.2 µg/g wet weight (WW) for whole body fish (0.31 µg/g WW in muscle) recommended to protect fish from injury. Mercury is a neurotoxin and endocrine disruptor, and impacts to fish may include reduced ability to avoid predators, secure food, and reproduce. The highest mercury concentrations were found in both Colorado pikeminnow and roundtail chub collected from the White River, a tributary to the Green River. Colorado pikeminnow from the White and Green rivers had the highest mean mercury concentrations and the lowest mean relative body conditions. Exposure to high mercury concentrations may act in concert with other threatening factors to compromise Colorado pikeminnow population viability and eventual recovery.

Water quality and fish surveys conducted during the past 20 years revealed widespread mercury contamination, especially in freshwater systems of the northern hemisphere (Schmidt and Brumbaugh 1990; Brumbaugh et al. 2001; Scudder et al. 2009; Cladis et al. 2014; Eagles-Smith et al. 2014, 2015, 2016). Although mercury occurs naturally in the environment and can be emitted from volcanic activity, and reemitted from oceans and forest fires, anthropogenic mercury emissions now far surpass those derived from natural processes (Mason and Sheu 2002; Pacyna et al. 2010; Driscoll et al. 2013; UNEP 2013). Inorganic mercury is released from industrial and energy facilities and during mining/metal processing (Eagles-Smith et al. 2016). In western North America, atmospheric mercury from transpacific transport (i.e., Asia) combines with local releases of inorganic mercury (Pacyna et al. 2010; Pirrone, et al. 2010; UNEP 2013) and returns to the landscape by wet or dry deposition. Mercury deposits enter waterways via watershed runoff, often ending up in lake bottoms and riverine wetlands (Lindberg et al. 2007; Peterson et al. 2007; Eagles-Smith et al. 2014, 2015, 2016).

After inorganic mercury enters aquatic ecosystems, conversion to methyl mercury is a key mechanism affecting bioaccumulation in the aquatic food web (Cocca 2001; Engstrom 2007; Pasquale et al. 2009; Tsui et al. 2010; Driscoll et al. 2013; Eagles-Smith et al. 2016). Fish accumulate methyl mercury mostly from their diet, and ingested mercury initially accumulates in fish intestines but then is transferred to other tissues, including blood, spleen, kidney, liver, and brain (Boudou et al. 1991; Wiener and Spry 1996; Sandheinrich and Wiener 2011). Skeletal muscle tissue is the primary ‘receiver’ of redistributed methylmercury. Once there, mercury forms a complex with protein (Sandheinrich and Wiener 2011) and is eliminated slowly, with an estimated half-life of approximately 400 days (Gonzalez et al. 2005) to 2 years (Wiener and Spry 1996).

Mercury in fish muscle is predominately methylmercury, so total mercury often is used as a surrogate measurement (Driscoll et al. 2013). Skeletal tissue concentrations vary with fish species, location, feeding habitats, and age (Sandheinrich and Wiener 2011). With food web biomagnification, fish at higher trophic levels usually contain the greatest mercury concentrations (Beckvar et al. 2005; Peterson et al. 2007; Sandheinrich and Wiener 2011).

Biotic and abiotic factors affect mercury toxicity in aquatic organisms. Environmental conditions (e.g., pH and temperature), sensitivities of individual species and life stages, and chemical and physical form of mercury, all affect toxicity (Wiener and Spry 1996). For several years, the scientific and regulatory focus on mercury in aquatic systems was motivated by the health risks to humans from consumption of mercury laden fish (Wiener and Spry 1996; USEPA 2001). However, several field and laboratory studies have demonstrated neurotoxic effects and impaired reproduction occur in the fish themselves at relevant dietary exposures similar to those found in the environment (Crump and Trudeau 2009).

The Colorado pikeminnow (*Ptychocheilus lucius*) evolved as the Colorado River Basin’s top predatory fish. As with all long-lived piscivores, Colorado pikeminnow are at risk of accumulating high mercury concentrations. Before the 1850s, they were abundant throughout warm-water reaches of the Colorado River Basin (Seethaler 1978; Platania 1990). By the 1970s, all lower basin populations (downstream of Glen Canyon Dam) and some upper basin populations were extirpated due to environmental alterations, including extensive dam-building (Miller 1961). The species was federally listed as endangered in 1967 (USFWS 1967; Miller 1961; Moyle 1976; Tyus 1991; Osmundson and Burnham 1998). Habitat considered critical to the survival and recovery of Colorado pikeminnow was later designated in portions of Colorado, Utah, New Mexico, Arizona, and California (USFWS 1994) (Fig. 1).

**Fig. 1:**
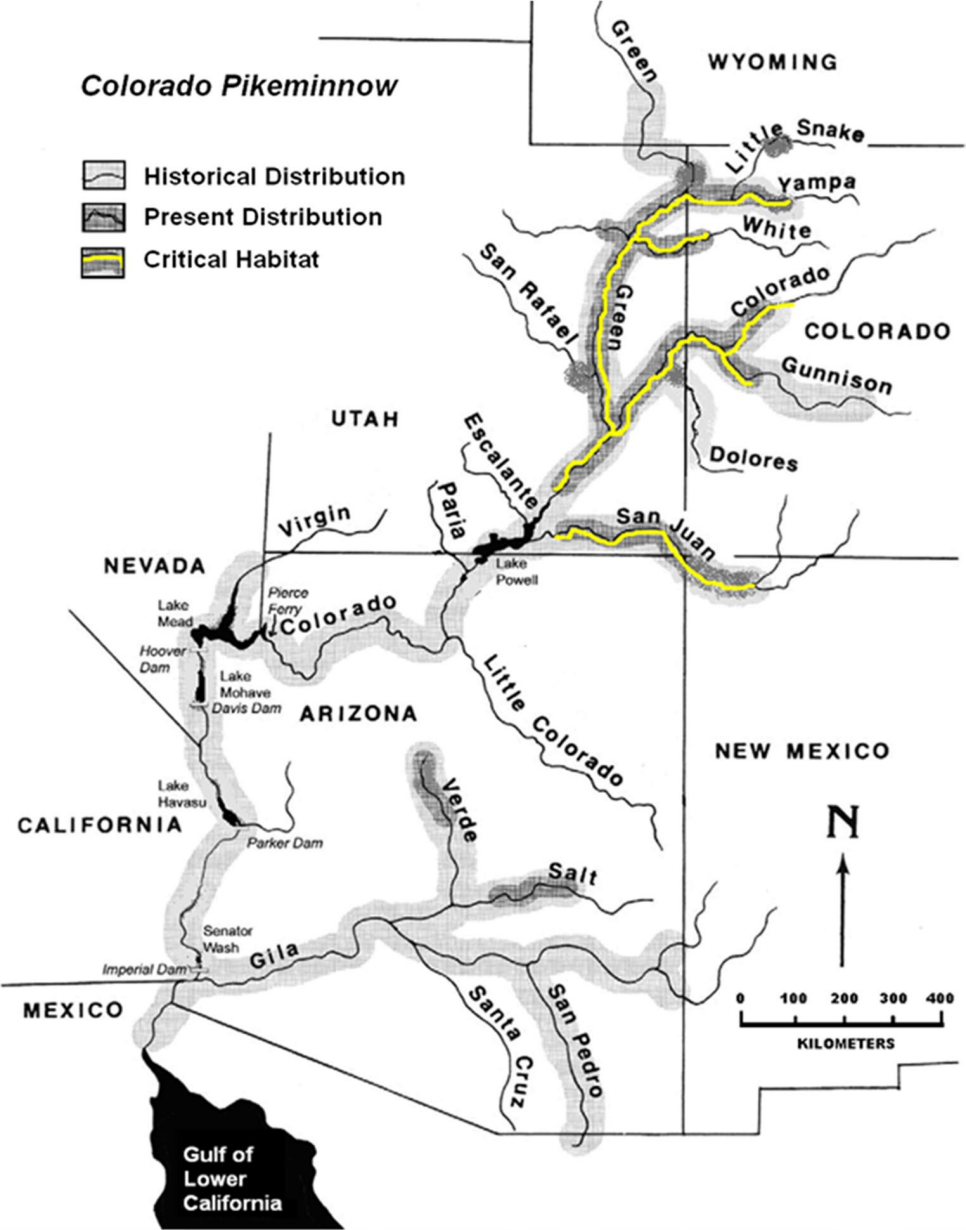
Distribution and critical habitat of the Colorado pikeminnow in the Colorado River system (USFWS 2014a, b)

Numerous sources of mercury emissions operate either adjacent to or upwind of critical habitat in Colorado, Utah, New Mexico, and Nevada (United States Environmental Protection Agency [USEPA] 2017). These sources of mercury, listed in USEPA’s (2017) TRI Explorer database, include: coal-fired power plants, copper smelters, gold ore processing with autoclave roasters, oil refineries, and cement and asphalt plants. These local emissions contribute several hundred pounds of atmospheric mercury annually in addition to that contributed by trans-Pacific sources (Seigneur et al. 2004).

Weidner (2007) and Sather et al. (2013) described mercury concentrations in precipitation at a monitoring location in southwestern Colorado as among the highest in the United States. Walters et al. (2015) found high mercury and selenium concentrations in fish food webs from the Colorado River in the Grand Canyon. Mercury contamination has been found in several fish species in the upper Colorado River basin. Six of 23 roundtail chub (*Gila robusta*) collected in 1992 from the Gunnison and Colorado rivers had concentrations > 0.3 mg/kg WW (Butler et al. 1994, 1995). A 2003 investigation revealed elevated concentrations in some smallmouth bass (*Micropterus dolomieu*) (0.25 ± 0.03 µg/g WW) collected from Colorado’s Yampa River and channel catfish (*Ictalurus punctatus*) (0.21 ± 0.00 µg/g WW) from Utah’s Green River (Hinck et al. 2006, 2007). Advisories for consumption of sport fish species have been posted within Colorado pikeminnow critical habitat in both Utah and Colorado. Seven Colorado pikeminnow that died in captivity (held as broodstock) 2–8 months after being taken from the White and Colorado rivers in 1986 were frozen, and their whole bodies later analyzed for mercury. Elevated concentrations were found in the four from the White River (0.31–0.96 µg/g WW) and in the three from the Colorado River (0.28–0.52 µg/g WW) (Krueger 1988).

Some investigators have suggested that mercury toxicity in fish might be counteracted by selenium, especially when Se:Hg molar ratios exceed 1 (Peterson et al. 2009; Penglase et al. 2014). These researchers suggest that presence of selenium in fish tissue must be considered when assessing potential toxicity of mercury concentrations. Contaminant analyses in fish tissue integrates the route, duration, and magnitude of exposure, as well as chemical form, metabolic transformations, and modifying biotic and abiotic factors. Mercury in fish tissue can be accurately assessed by using muscle biopsies (Pearson 2000; Baker et al. 2004; Peterson et al. 2005). To do so nonlethally (i.e., for endangered species), a dermal muscle punch can be used to sample tissue for accurate assessment of mercury residues. To determine if mercury exposure could be a factor contributing to the decline of this species, or perhaps hampering recovery efforts, we assessed mercury concentrations in muscle tissue of Colorado pikeminnow sampled from throughout their remaining range. We also assessed mercury concentrations in roundtail chub sampled in the White River. Our objectives were: (1) to determine whether concentrations exceeded established guidelines for fish health; (2) to determine whether mean concentrations varied by fish size and by river; (3) to determine Se:Hg ratios; and (4) to assess whether body weight (an index of fish health) is affected by mercury concentrations.

## Materials and Methods

### Study Area

Muscle tissues were collected from wild Colorado pikeminnow captured from 330 river kilometers (rkm) of the Green River, 122 rkm of the Colorado River, 164 rkm of the White River, and 34 rkm of the Yampa River (Fig. 1; Table 1). Some also were collected from individuals in the lowermost 3.7 rkm of the Gunnison River (downstream of Redlands Diversion Dam), comprising part of the Colorado River population (Osmundson et al. 1998). Captures of stocked, hatchery-reared, Colorado pikeminnow were from 64 rkm of the San Juan River. All reaches sampled were within critical habitat and represented much of the species’ remaining range.

**Table 1:** River, river segment, river kilometer locations, segment description, and Universal Transverse Mercator (UTM) coordinates of river segment where Colorado pikeminnow were captured in 2008 and 2009

River	River segment and (# of fish captured)	River kilometer locations (converted from river miles)	Segment description	UTMs (Zone 12; meters East (E) and North (N)
Colorado (23)	Lower Colorado (12)	68.1–170.3	Below Potash to Fish Ford launch	615674 E 4255628 N–652223.5 E 4309671 N
	Upper Colorado (11)	275–295	Gunnison River confluence to Labor Camp	710178.1 E 4325780 N–726547 E 4330627 N
San Juan (29)		170.7–235.5	Aneth to Shiprock	665398 E 4114487 N–703649 E 4074289 N
White (10)		1.5–165	Close to Green River confluence to Below Taylor Draw Dam	636766 E 4433366 N–667791 E 4430780 N
Yampa (8)		131.4–166.5	Upper Maybell to Morgan Gulch	748673 E 4489051 N–Zone 13 256857 E 4473396 N
Green (29)	Lower Green (10)	86.6–178.9	Green River, Utah	586913 E 4267030 N–584974 E 4273276 N
	Middle Green (9)	214.9–383.7	Close to Price River confluence Below Ouray	581269 E 4326476 N–631382 E 4429511 N
	Upper Green (10)	468.5–537.6	Bonanza Bridge to Island Park	631382 E 4463388 N–656798 E 4485121 N

### Muscle Tissue Collection

Muscle plugs were collected from 41 Colorado pikeminnow during late spring 2008 and 57 during 2009 (Table 1). In 2008, 29 samples were from upper, middle, and lower Green River reaches, 8 from the Yampa River, and 4 from the White River. In 2009, 23 samples were collected from the lower and upper Colorado River reaches, 29 from the San Juan River, and 6 from the White River. Muscle plugs also were taken from 11 roundtail chubs captured from the White River in eastern Utah during 2009. Muscle tissue collection followed procedures of Williamson (1992), Baker et al. (2004) and Peterson et al. (2005) with some modifications. Fishery crew members from federal and state agencies, and Colorado State University, were first instructed to follow standardized procedures for safe handling and length measuring of fish and collecting and preserving muscle tissue. A 5-mm dermal punch was inserted 1–2 cm below the dorsal fin, and a plug of muscle tissue was extracted with a slight twisting motion. Breaking off of the tissue sample was facilitated by tilting the punch during removal. A different punch was used on each fish and discarded after use. A single muscle plug was taken from each fish and placed in an acid-washed cryogenic vial. These were kept on wet ice until crews returned from the field. Betadine was applied to the fishes’ wound to decrease the risk of infection and promote healing. Samples were frozen upon return from the field. After all samples were collected, muscle plug skins were dissected and removed using acid-washed, stainless steel surgical equipment. Tissues were then returned to containers and frozen to − 20 °C. Samples were inventoried and shipped to the Trace Element Research Laboratory (TERL) in College Station, Texas, for mercury analysis using combustion atomic absorption spectrometry (CAAS) (USEPA 1998; Cizdziel et al. 2002).

### Mercury Toxicity Guidelines

In 2001, the U.S. Environmental Protection Agency developed a methyl mercury water quality criterion of 0.3 mg/kg wet weight (WW) in edible fish tissue, designed for the protection of humans consuming fish (USEPA 2001). Regarding the protection of the health of fish themselves from mercury, various authors have suggested different tissue toxicity threshold-effect levels. In a review of mercury toxicity to freshwater fish, Sandheinrich and Wiener (2011) reported fish-tissue mercury concentrations starting at 0.3 µg/g WW in whole body fish and 0.5 µg/g WW in fish axial muscle were associated with effects on biochemical processes, damage to cells and tissues, and reduced reproduction. After reviewing 10 mercury residue-effect studies for 8 fish species, and using survival, growth, reproduction, and behavior as toxicity endpoints, Beckvar et al. (2005) recommended a 0.2 µg/g mercury whole body guideline to protect juvenile and adult fish health. Dillon et al. (2010) developed a mercury dose–response curve based on published fish tissue-residue toxicity studies, in which endpoints related to mortality (i.e., survival and reproductive success) were selected. They found residues of 0.1 µg/g WW associated with a 2.8% injury rate (e.g., reduced survival) in adult fish and a 19.8% injury rate in early life stages. Higher mercury concentrations were associated with higher levels of injury. Because Colorado pikeminnow are endangered, we chose the more protective mercury toxicity threshold of 0.2 µg/g WW in whole body fish (Beckvar et al. 2005) as a standard with which to compare our results.

Regression equations developed for various fish species have been used to convert mercury concentrations in skinless muscle tissue to whole body concentrations (Peterson et al. 2005). One equation developed for Northern pikeminnow (*Ptychocheilus oregonensis*), a physiologically similar species to Colorado pikeminnow, had a slope of 0.9048 and intercept of −0.2387. We used this equation to convert the recommended whole body toxicity threshold of 0.2 µg/g WW (Beckvar et al. 2005) to a corresponding muscle tissue toxicity threshold of 0.31 µg/g WW.

### Statistical Analysis and Data Interpretation

1. Colorado pikeminnow mercury concentrations were transformed (Log_10_(X + 1)) to improve normality and *t* tests (*p* < 0.05) were then used to compare mercury concentrations among rivers
2. Relations between body size and mercury concentration were investigated with regression, using transformed (Log_10_) fish total lengths (independent variable) and transformed (Log_10_(X + 1)) muscle plug mercury concentrations (dependent variable).
3. We regressed transformed (Log_10_) Colorado pikeminnow total length and mass measurements and used the slope and y-intercept to calculate relative body condition.
4. Relative condition (*K*_n_) of Colorado pikeminnow was calculated using the following equations from Osmundson and Burnham (1998):
  1 $$K_{\text{n}} = 100 \, \times \, M_{\text{o}} /M_{\text{e}}$$
  where *M*_o_ is the observed mass (g) and *M*_e_ is the expected mass (g) as calculated from:
  2 $$\log_{10} \left( {M_{\text{e}} } \right) \, = \, \log_{10} \left( {\text{length}} \right)*m + \, b$$
  *M*_e_ was calculated from transformed lengths and weights of Colorado pikeminnow sampled for muscle tissues from all river reaches, with (*m*) as the slope and (*b*) as the y-intercept (developed under #3 above).
5. Mean *K*_n_ of Colorado pikeminnow was compared among rivers and relative conditions of individual fish (dependent variable) were regressed against the corresponding mercury concentrations found for those fish (independent variable) to assess whether mercury burdens might affect fish body condition.
6. Mass concentrations of selenium and mercury were converted to molar concentrations by dividing by their molecular weights (Se = 78.96; Hg = 200.61; Peterson et al. 2009). We compared molar concentrations of Se:Hg to estimate when and where any ‘surplus’ selenium was potentially available for protection against mercury toxicity. Mean selenium concentrations for Colorado pikeminnow sampled during 1996 in the same river segments as this study (listed by Hamilton et al. 2003) were used for comparisons with our more recently observed mercury concentrations.
7. Mercury concentrations in Colorado pikeminnow muscle plugs were compared to those of other fish species reported to have adverse biological effects (i.e., converted guideline for muscle tissue of 0.31 µg/g WW per Beckvar et al. (2005).

## Results

### Mercury Concentrations

The mean mercury concentration in muscle plugs from 99 Colorado pikeminnow from combined river segments was 0.49 µg/g WW (95% confidence interval [CI] = 0.42–0.56), exceeding the recommended fish muscle toxicity guideline for fish health (0.31 µg/g WW). The mean concentration for 83 Colorado pikeminnow > 400-mm long was 0.66 µg/g WW (95% CI = 0.6–0.72) or twice that of the toxicity guideline. For the 10 Colorado pikeminnow sampled from the White River, the mean concentration (1.1 µg/g WW (95% CI = 0.79–1.31) was over three times that of the toxicity guideline (Table 2). This mean concentration in White River samples was significantly higher than those means from the Colorado and Yampa river segments and likely would have been significantly higher than that in Green River Colorado pikeminnow (*α* = 0.06) had the sample size been larger. For the 11 White River roundtail chubs, the mean concentration (0.59 µg/g/WW [95% CI = 0.42–0.60]) also exceeded the toxicity guideline.

**Table 2:** Summary statistics for mercury concentrations (wet weight) in muscle plugs taken from Colorado pikeminnow (CPM) and roundtail chub (RTC) in river segments in critical habitats for Colorado pikeminnow. The geometric mean for fish total length for each river segment also is provided

Matrix	Statistic	Green River	Yampa River	White River		Colorado River	San Juan River	
				Colorado pikeminnow	Roundtail chub		< 400 mm total length	> 400 mm total length
Mercury	Median	0.72	0.49	1.02	0.52	0.56	0.09	0.38
	Geometric mean (95% CI’s)	0.7 (0.66–0.74)	0.48 (0.45–0.51)	1.01 (0.88–1.15)	0.57 (0.3–0.89)	0.57 (0.51–0.61)	0.11 (0.09–0.13)	0.37 (0.32–0.42)
	Minimum	0.32	0.39	0.43	0.11	0.31	0.03	0.31
	Maximum	1.08	0.58	1.8	1.97	1.04	0.28	0.43
	*n*	29	8	10	11	23	26	3
Total length	Geometric mean (95% CIs)	501 (479–525)	589 (552–604)	562 (513–617)	282 (246–324)	599 (549–649)	282 (269–295)	427 (418–442)
Body condition	Mean (95% CIs)	96 (90–102)	117 (108–126)	88 (82–94)		105 (98–111)		
CPM over 400 and RTC over 250 mm Total length that exceed toxicity GL		28/29	8/8	10/10	6/11	23/23	0/26	2/3

**Fig. 2:**
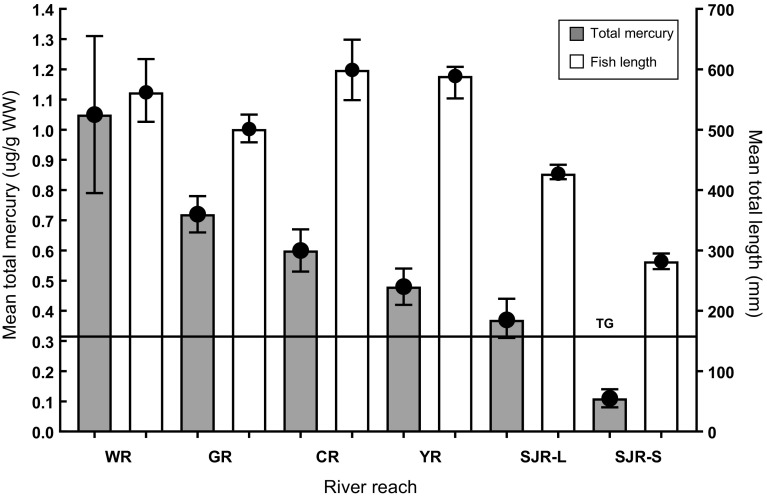
Mean (+ 95% CI) total mercury and mean fish total length for Colorado pikeminnow from each river segment in critical habitat. Rivers include: *WR* White River, *GR* Green River, *CR* Colorado River, *YR* Yampa River, *SJR*-*L* San Juan River fish > 400 mm total length, *SJR*-*S* San Juan River fish < 400-mm total length. Horizontal line = toxicity guideline (TG) of 0.31 µg/g WW

For samples from combined Green River reaches, the mean concentration was 0.72 µg/g WW (95% CI = 0.66–0.78), for samples from combined Colorado River reaches, 0.6 µg/g WW (95% CI = 0.53–0.67), and from the Yampa River, 0.48 µg/g WW (95% CI = 0.43–0.53) (Table 2; Fig. 2). Many of the stocked Colorado pikeminnow in the San Juan River were < 400-mm long, and those that were larger had concentrations exceeding the toxicity guideline. Aside from means, mercury concentrations exceeded toxicity thresholds in most individual Colorado pikeminnow sampled, particularly those from the White River.

### Fish Length and Mercury Concentration

**Fig. 3:**
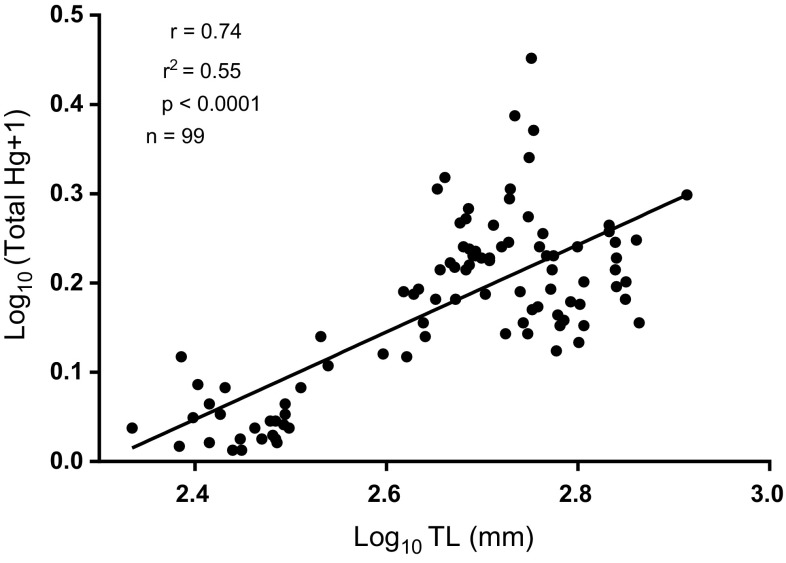
Log-transformed total mercury concentration, Log_10_([T-Hg] + 1), versus log-transformed total length, Log_10_(TL) for all sampled Colorado pikeminnow from combined river segments'

To determine if mercury concentrations differed among river segments because of size differences in sampled fish, we compared mean total lengths among fish from each river segment (Table 2; Fig. 2). Colorado pikeminnow from the Yampa River were some of the largest fish captured but had lower mercury concentrations than those from other rivers. Mercury concentrations were highest in White River Colorado pikeminnow, but these fish were not significantly larger than those captured in the Colorado and Yampa rivers. Those White River Colorado pikeminnow with the highest overall mercury concentrations (> 1.0 µg/g WW) were not the largest fish captured during the study. Hence, there appeared to be a river effect separate from any length effect. However, there was a significant correlation (*r*^2^ = 0.65, *p* = 0.0000) between mercury concentration and total length when samples from all river segments were pooled (Fig. 3).

Of roundtail chubs from the White River, the largest individuals (almost 400-mm long and > 400 g) contained the highest mercury concentration (almost 2.0 µg/g WW). In addition, there was a high and significant correlation (*r*^2^ = 0.8, *p* < 0.001) between mercury concentration and roundtail chub length.

### Body Condition and Mercury Concentration

**Fig. 4:**
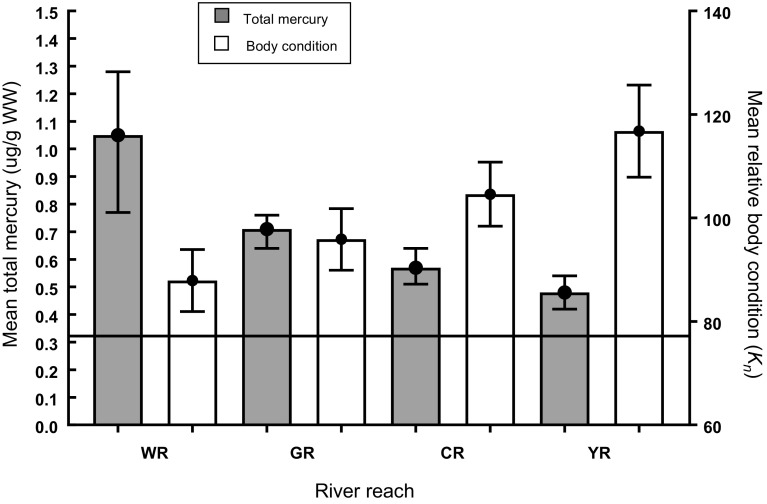
Mean (+ 95% CI) total mercury and relative body condition (*K*n) for Colorado pikeminnow sampled from the White, Green, Colorado, and Yampa rivers. River reaches include: *WR* White River, *GR* Green River, *CR* Colorado River, *YR* Yampa River. Horizontal line = toxicity guideline of 0.31 µg/g WW

**Fig. 5:**
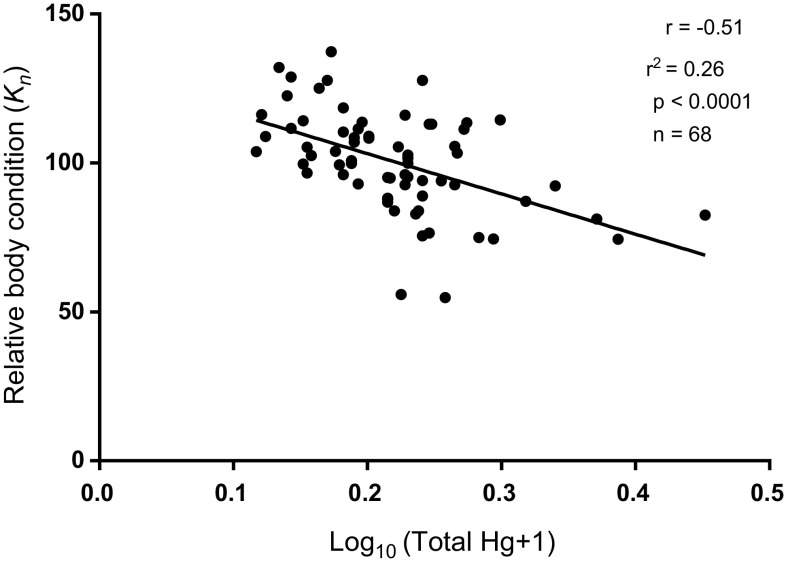
Relative body condition versus log-transformed total mercury concentrations, Log_10_ ([T-Hg] +1), for Colorado pikeminnow sampled from the White, Green, Colorado, and Yampa rivers

Because most Colorado pikeminnow from the San Juan River were relatively small and recently stocked before capture, they were not included in body condition analyses. Length and weight of Colorado pikeminnow from the White, Green, Yampa, and Colorado rivers were strongly correlated variables (*r*^2^ = 0.94, *p* < 0.0001, *n* = 68), as expected. Length–weight regression coefficients, used in calculating relative body condition, had a slope (*m*) of 3.19111 (SE = 0.104) and y-intercept (b) of −5.587 (SE = 0.154). Colorado pikeminnow from the White and Green rivers had the highest mean mercury concentrations and the lowest mean relative body conditions (Fig. 4), whereas those from the Yampa and upper Colorado rivers had lower mean mercury concentrations and higher mean relative body conditions. When Colorado pikeminnow were pooled (*n* = 63) from all sites (excluding the San Juan River), there was a significant (*p* < 0.0001) trend (*r*^2^ = 0.26) and inverse correlation (*r* = − 0.51) between mercury concentration and relative body condition (Fig. 5).

### Selenium–Mercury Interaction

Colorado pikeminnow from all river segments had higher mass and molar selenium concentrations than mercury concentrations (Table 3), based on mean concentrations from two separate sampling periods. Surplus selenium, which may afford protection against mercury in Colorado pikeminnow, was highest in the Colorado River and lowest in the Yampa and White rivers (Table 3).

**Table 3:** Mass and molar concentrations of mercury (Hg) and selenium (Se) and surplus selenium concentrations in Colorado pikeminnow (selenium concentrations are from Hamilton et al. 2003)

	Mean mercury concentration (minimum, maximum)		Mean selenium concentration (minimum, maximum)		Surplus Se µmol/g Se
	µg/g Hg WW	µmol/g Hg WW	µg/g Se WW	µmol/g Se WW	
*River Segment*					
Mid Green R.	0.77 (0.68,0.87)	0.0038	0.98 (0.87, 1.08)	0.0124	0.0086
Yampa R.	0.49 (0.44, 0.53)	0.0024	0.62 (0.44, 0.72)	0.0079	0.0055
White R.	0.95 (0.43, 1.83)	0.0052	0.93 (0.64, 1.18)	0.0118	0.0071
San Juan R. (> 400 mm)	0.236 (0.199, 0.267)	0.0007	0.83 (0.74, 1.0)	0.0105	0.0098
Colorado R.	0.6 (0.31, 1.04)	0.003	1.92 (0.93, 2.16)	0.0243	0.0214

## Discussion

### Difference in Mean Mercury Concentrations Among Rivers

Elevated mercury concentrations reported here verify not only that mercury deposition occurs in watersheds containing Colorado pikeminnow critical habitat but also that mercury has moved through the aquatic food web and into the tissues of roundtail chub and endangered Colorado pikeminnow. Sources are not definitively known, but deposition likely includes both local and global contributions. Following deposition, conditions favoring methylation of inorganic mercury enable bioaccumulation. Our results suggest that remaining populations of Colorado pikeminnow are at risk of suffering the adverse biological effects associated with mercury exposure.

The Yampa and White rivers in northwestern Colorado are relatively close geographically and run roughly parallel to one another. Because of this, aerial (wet and dry) deposition rates into each river was expected to be similar. Yet Colorado pikeminnow from the Yampa River had the lowest concentrations of the study (excluding recently stocked San Juan River fish), whereas those in the White River had the highest concentrations. This suggests a localized source(s) of mercury entering the White River. Landscape modifications, including land disturbance, can alter mercury inputs to downstream aquatic ecosystems (Evers et al. 2007). Other localized sources might include mining or other geochemical, biological, or watershed factors affecting levels of mercury bioaccumulation. Landscape characteristics influencing mercury transport to surface waters include land cover, oxidation–reduction conditions, hydrologic flow paths, large water-level manipulations in reservoirs, and nutrient loading (Evers et al. 2007). Further detailed comparisons of the Yampa and White River basins are needed to help explain the differences in mercury burdens noted here.

### Fish Length and Mercury Concentration

Mercury in fish tissue may increase most rapidly when fish are small and growing quickly, particularly when piscivorous species shift from a diet of invertebrates to one of other fish. Mercury concentrations in fish tissue generally rise with increasing fish age or body size because of the slow rate of elimination relative to the faster rate of uptake (Sandheinrich and Wiener 2011; Wiener and Spry 1996). Colorado pikeminnows switch from an insectivorous to a piscivorous diet when they exceed 40- to 100-mm long (Vanicek and Kramer 1969; Muth and Snyder 1995). Diet studies of Colorado pikeminnow in the Colorado River indicated that individuals 400- to 550-mm long had primarily eaten other fish species, including roundtail chubs, and larger Colorado pikeminnow ate larger prey fish (Osmundson et al. 1998). As results from this study demonstrate, mercury muscle concentrations increase with Colorado pikeminnow size. Large individuals are older and thus have accumulated mercury for a longer period. Also, because they eat larger prey than do smaller Colorado pikeminnow, larger prey fish themselves may have higher mercury burdens. For White River roundtail chub, there also was a positive and significant correlation between mercury concentration and fish length, and the largest roundtail chubs contained concentrations as high as those found in Colorado pikeminnow. Other factors, as yet unknown, also influence tissue concentrations and are evidently specific to the river segment in which each fish primarily resides.

### Fish Body Condition and Mercury Concentration

Body condition in fish often is used to gauge the overall health of an individual fish. Relative condition factor is an index that relates an individual’s plumpness to that of the average fish in the population or in many populations combined. Thus, it indicates the degree of difference in the observed weight of a fish compared with the species-specific expected weight for a fish of its length (Le Cren 1951). Reduced body condition associated with mercury exposure has been found in various fish species, including striped bass (*Morone saxatilis*), Northern pike (*Esox lucius*), white sturgeon (*Acipenser transmontanus*), and walleye (*Stizostedion vitreum*) (Sandheinrich and Wiener 2011). This inverse relationship between mercury concentration and body condition, however, can be confounded and complicated by other co-occurring contaminants or by other environmental factors. Osmundson et al. (1998) reported that mean *K*_n_ of Colorado pikeminnow in the Colorado River significantly decreased with increased fish size in the lower river segment but increased with fish size in the upper river segment. In addition, mean *K*_n_ within several 100-mm length classes significantly differed among four 3-year study periods (Osmundson and White 2014). Those investigators attributed spatial and temporal changes in mean *K*_n_ to variation in food availability. Our results indicate mercury exposure is also affecting Colorado pikeminnow body condition. Mean *K*_n_ was lowest in Colorado pikeminnow from the White and Green rivers, where mercury residues were highest. In addition, we found a significant inverse correlation between body condition and mercury concentration when individuals from four rivers were pooled. The *r*^2^ value (0.26, *p* < 0.0001) was not high, but this is expected given that food availability, water temperature, and other environmental variables also affect condition. Most importantly, reduced body condition associated with elevated mercury residues points to mercury negatively impacting the health of these fish.

### Selenium–Mercury Interaction

Accumulations of selenium and mercury individually can result in irreversible injury during early development of fish (Mailman et al. 2014; Walters et al. 2015). Some researchers have reported evidence of selenium moderating the negative effects of mercury in freshwater fish when accumulated together (Peterson et al. 2009), but underlying mechanisms and the amount of excess selenium needed are unclear (Khan and Wang 2009). Kim et al. (1977) and Cuvin and Furness (1988) demonstrated both antagonistic and synergistic toxic interactions between selenium and mercury are possible and are a function of concentrations and the molar ratio of one to the other. More recently, low-level addition of selenium to a lake was found to decrease MeHg bioaccumulation in fish gonads (Mailman et al. 2014). The optimal antagonistic molar ratios for selenium and mercury in the environment or in tissues (along with other contaminants and environmental stressors) have not yet been determined for Colorado pikeminnow.

In 468 fish, representing 40 western U.S. species, 97.5% had a molar ratio > 1 (i.e., containing more selenium than mercury) (Peterson et al. 2009). Fish species with Se:Hg < 1 included other pikeminnow species. Colorado pikeminnow from the White River had the highest mercury concentrations observed during this study and therefore are at greatest risk of injury from mercury exposure. The role that selenium may play in counteracting these effects is currently unknown.

### Mercury Levels in Colorado pikeminnow and Those in Other Species Demonstrating Toxicity

More than half of the Colorado pikeminnow sampled for this study exceeded the 0.2 µg/g WW whole body fish toxicity threshold (0.31 µg/g WW in fish muscle) proposed by Beckvar et al. (2005). A residue-based mercury dose–response model for fish (Dillon et al. 2010) associates a mercury residue of 0.3 µg/g WW whole body (= 0.45 µg/g WW in muscle) with an 8% injury rate for juvenile/adult fish and a 42.5% injury rate for early life stages. Comparison of mercury levels in Colorado pikeminnow to this dose–response curve suggests that many individuals probably experience some rate of impaired reproduction, reduced growth, and reduced survival.

A population viability analysis recently modeled for Colorado pikeminnow in the San Juan River estimated that current levels of mercury toxicity would reduce reproductive success by 2% among newly recruited adult females (Miller 2014). As these females age, the percent injury was expected to increase to 5%. If mercury deposition in the San Juan River increases in the future as anticipated, injury estimates are predicted to increase to 3.5–9%. Under this assumption, the estimated injuries to both reproductive success and age-specific survival led to decreases in simulated population growth potential (Miller 2014). Thus, anticipated mercury load increases in the San Juan sub-basin are expected to reduce the effectiveness of current recovery efforts.

Sublethal effects can be important in fish. Crump and Trudeau (2009) reported that mercury accumulation in the fish brain resulted in reduced hormone secretion, nerve damage, and alterations in neurotransmission. As a neurotoxin, mercury can alter behavior, reducing predator avoidance ability and/or the ability to secure food, leading to slow growth and emaciation (Sandheinrich and Atchison 1990; Webber and Haines 2003; Crump and Trudeau 2009; Sandheinrich and Wiener 2011; Depew et al. 2012). Lowered body condition, as noted, would be an expected result of this. Mercury also can negatively affect fish endocrine systems, resulting in diminished reproductive success (Matta et al. 2001; Hammerschmidt et al. 2002; Drevnick and Sandheinrich 2003; Tan et al. 2009; Sandheinrich and Wiener 2011; Richter et al. 2014). Crump and Trudeau (2009) suggested that the inhibitory effect of mercury on reproduction in fish may occur at multiple sites within the reproductive system, including the hypothalamus and pituitary in the brain, in the gonads themselves, and in other tissues and functions of the endocrine system. Investigations into the effects on reproductive organs demonstrated a range of injuries, including reduced gonad size and gamete production, reduced circulating reproductive steroids, and lowered spawning success (Crump and Trudeau 2009; Tan et al. 2009; Sandheinrich and Miller 2006; Sandheinrich and Wiener 2011).

Higher trophic level fish can accumulate substantial tissue mercury, and females can transfer it to developing eggs during oogenesis (Hammerschmidt and Sandheinrich 2005; Alvarez et al. 2006; Crump and Trudeau 2009). In addition to maternal transfer, eggs released into the aquatic environment may accumulate mercury directly from the water (Crump and Trudeau 2009). Beckvar et al. (2005) and Dillon et al. (2010) suggested that early life stage fish have a much greater sensitivity to mercury compared with juvenile and adult fish. Dillon et al. (2010) calculated a median effect concentration (MC50) for mercury in larval fish to be 0.406 µg/g WW (95% CI = 0.117–1.405 µg/g WW). Behavioral impairment after developmental mercury exposure has been demonstrated in laboratory studies (Fjeld et al. 1998; Alvarez et al. 2006; Weber 2006) at mercury concentrations as low as 0.007–0.015 µg/g WW (Weber 2006). Behavioral impairments include reduced competitive feeding ability and predator-evasion responses, which in turn may reduce survival and recruitment to endangered fish populations.

Crump and Trudeau (2009) also discussed a mercury-altered lipid balance resulting in disruption of vitellogenesis and egg production. Richter et al. (2011) found individual genes (related to nervous system development and lipid metabolism) in female zebrafish (*Danio rerio*) exhibiting altered expression in response to methylmercury exposure. Similar tissue-level studies are needed for Colorado pikeminnow to assess degree of injury in individuals and what, if any, adverse impacts might be expected at the population level.

### Status of Colorado pikeminnow

The status of wild Colorado pikeminnow populations in all upper basin rivers remains tenuous. The San Juan River population consists almost exclusively of stocked fish, the last capture of a wild adult having occurred in 2000 (Ryden 2003; Furr and Davis 2009; Durst and Franssen 2014). At least some of the stocked fish have survived to maturity and the presence of larvae in the system verifies successful reproduction has occurred. However, recruitment of these wild-produced larvae to the adult phase has not yet been documented (Durst and Franssen 2014).

A recent decline in wild adults has been reported for the Green River basin (Bestgen et al. 2016a, b) as well as the upper Colorado River (Osmundson and White 2017; Elverud and White 2017). Especially concerning is the very weak age-0 representation in the middle Green River from 1999 to 2013 (Bestgen and Hill 2015; Bestgen et al. 2016a). A similar decline in young of the year fish (YOY) in the Colorado River from 1997 to 2013 led researchers to conclude that recruitment rates have been insufficient to offset adult mortality rates (Osmundson and White 2017).

Water regulation is thought to negatively affect reproductive success and habitat suitability for young (Bestgen and Hill 2015; Osmundson and White 2017), while persistently high densities of nonnative predators (e.g., smallmouth bass (*Micropterus dolomieu*), northern pike, and walleye), particularly in the Yampa River (Johnson et al. 2008; USFWS 2014a, b, 2015), reduce survival of juveniles. In combination, rates of recruitment to the adult stage are depressed. Predation by nonnative fish on young Colorado pikeminnow is an obvious, direct cause of mortality, while river regulation and the pathways by which associated impacts affect reproduction and recruitment are more complex and therefore less well understood. Layered on this are the effects of environmental contaminants. Our results, along with those from studies of other fish, suggest mercury burdens are sufficiently high in Colorado pikeminnow that negative effects at the population level should be expected.

## Conclusions

High mercury concentrations are known to adversely affect reproductive output and adult survival in fishes. For Colorado pikeminnow, the high concentrations documented here may act multiplicatively with other threats to reduce population growth rate and ultimately impact recovery potential. Mercury exposure was found in all sampled Colorado pikeminnow. Those > 400-mm long contained mercury above recommended toxicity guidelines designed to protect demographic endpoints, such as reproduction and survival. Although the role that selenium may play in counteracting mercury toxicity is unknown, the relationship we found between mercury concentration and reduced body condition strongly suggests that injury is occurring. Tissue-level studies are needed to better understand physiological pathways of impairment and quantify toxicity effects. Managers tasked with restoring sustainable Colorado pikeminnow populations need to consider mercury contamination as an important threat to demographic rates and recovery of Colorado pikeminnow. Collaborative efforts with regulatory agencies are needed so strategies to reduce sources of mercury can be developed.

Because mercury deposition is so widespread, specific fish species from throughout North America and around the globe are at risk of toxicity effects. Such effects may be insignificant for short-lived fishes or those that occupy a low trophic niche. However, for long-lived predatory fish, concentrations are expected to be high and can have deleterious effects at both the individual and population level. When such species are already endangered due to other impacts to their environment, contaminants, such as mercury, can impede efforts to improve reproduction and recruitment and therefore should not be ignored when developing recovery strategies.
